# CXCL16 Producing Tumor Clones Are Shaping Immunosuppressive Microenvironment in Squamous Cell Carcinoma via CXCR6 Regulatory T Cell

**DOI:** 10.1002/cam4.71060

**Published:** 2025-08-07

**Authors:** Hyun Seung Choi, Sunyoung Jung, Ki‐Myo Kim, Mihyun Lee, Jun Ho Park, Sanha Hwang, Seung Min Cha, Jade N. Young, Dina Poplausky, Hyunsung Nam, Nicholas Gulati, Chung‐Gyu Park, Hyun Je Kim, Ji‐Ung Park

**Affiliations:** ^1^ Department of Biomedical Sciences Seoul National University Graduate School Seoul the Republic of Korea; ^2^ Cancer Research Institute Seoul National University College of Medicine Seoul the Republic of Korea; ^3^ Department of Plastic and Reconstructive Surgery Seoul National University Boramae Hospital, Seoul National University College of Medicine Seoul the Republic of Korea; ^4^ Department of Dermatology Icahn School of Medicine at Mount Sinai New York USA; ^5^ Genomic Medicine Institute Seoul National University College of Medicine Seoul the Republic of Korea; ^6^ Transplantation Research Institute Medical Research Center, Seoul National University College of Medicine Seoul the Republic of Korea; ^7^ Department of Dermatology Seoul National University Hospital Seoul the Republic of Korea; ^8^ Interdisciplinary Program in Artificial Intelligence (IPAI), Seoul National University Seoul the Republic of Korea

**Keywords:** basal cell carcinoma, cutaneous squamous cell carcinoma, scRNA and TCR seq, spatial transcriptomic

## Abstract

**Background:**

Cutaneous squamous cell carcinoma (cSCC) and basal cell carcinoma (BCC) are the most prevalent types of nonmelanoma skin cancer (NMSC) and exhibit significant inter‐ and intra‐tumor heterogeneity. cSCC has a higher metastatic potential than BCC, accompanied by a considerable mortality rate. However, the detailed mechanisms of tumor evolution in cSCC have not yet been described.

**Methods:**

We performed single‐cell RNA sequencing (scRNA‐seq) and T cell receptor (TCR) clonal analysis of skin biopsies from five BCCs, three squamous cell carcinomas in situ (SCCIS), and two invasive squamous cell carcinomas (SCC). Independent SCC specimens were used for spatial transcriptomic (ST) analysis using GeoMx Digital Spatial Profiler (DSP).

**Result:**

Using scRNA‐seq, we analyzed a total of 117,663 cells. We distinguished cancer cells using copy number variation and identified SCC‐specific genes that potentially contribute to tumor progression. Analysis of tumor clones revealed SCC‐specific *COL6A1*+/*ITGA5*+ carcinoma cells which produce *CXCL16*. We also annotated *CXCR6*+ regulatory T cells (Tregs) which potentially move toward the tumor site by CXCL16, shaping the immunosuppressive TME. ST analysis supported these clones were located at the invasion site of SCC.

**Conclusion:**

We suggest COL6A1 and ITGA5 promote the invasive and metastatic property of SCC. We also uncovered how SCC recruits Tregs via the CXCL16/CXCR6 axis to create a TME favorable for its survival. These molecules can be used as potential therapeutic targets for treatment of SCC.

## Graphical abstract

SCC shapes an immunosuppressive TME via CXCL16/CXCR6 axis.

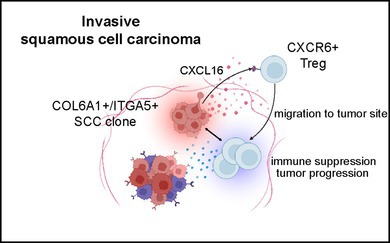

## 1 Introduction

Keratinocyte carcinomas including cSCC and BCC are the most common NMSC, and their incidences continue to increase [1, 2]. Although both cSCC and BCC originate from keratinocytes and primarily result from mutations induced by UV exposure [3], they exhibit distinct characteristics. While BCC is associated with uncontrolled activation of the hedgehog signaling pathway [4], cSCC is characterized by cumulative gene mutations affecting suppressor genes like *CDKN2A* and *NOTCH*, as well as oncogenes like RAS. Additionally, several signaling pathways, such as NF‐kB, MAPK, and PI3K/AKT/mTOR, play roles in the increased expression of the epidermal growth factor receptor (EGFR) [5, 6]. Surgical excision is commonly advised for both BCC and cSCC, resulting in high rates of successful treatment. In contrast to BCC, metastasis occurs in 1.2%–5% of patients with cSCC, leading to a 3‐year mortality rate of 46% for metastatic disease [4, 7]. Given the complexities of excision in these cases, alternative therapies are imperative. EGFR inhibitors and immunotherapy such as anti‐PD‐1 therapy are now established as standard care in advanced or metastatic cSCC [8]. Nevertheless, cSCC's pronounced heterogeneity, stemming from cumulative mutations, complicates patient stratification. Hence, identifying biomarkers for cSCC treatment requires conducting gene expression profiling analysis in each individual cSCC. Previous traditional bulk transcriptome sequencing (bulk RNA‐seq) has offered valuable insights into the genetic basis of BCC and cSCC [9, 10]. However, these approaches, involving a mixture of various cells, often conceal significant aspects of intra‐tumor heterogeneity, thereby limiting our insight into cSCC biology. The development of single‐cell RNA sequencing (scRNA‐seq) enables the elucidation of complex tumor diversity and dissects the dynamic communication network between the tumor and its microenvironment [11]. Utilizing scRNA, several groups have documented the gene expression patterns in human BCC and cSCC. Chistian defined BCC cellular heterogeneity and identified the heat shock protein (HSP) pathway as a potential therapeutic option for BCC [12]. Andrew and Li discovered a tumor‐specific keratinocyte (TSK) population unique to cSCC, serving as a hub for intercellular communication and exhibiting remarkable epithelial–mesenchymal transition (EMT) features [13, 14]. Despite this, deciphering both tumor heterogeneity and its interactions with surrounding cells through high‐resolution methods remains imperative. Here, we first characterize the differences between BCC and cSCC at the single‐cell transcriptomic level combined with spatial transcriptome and identify potential invasive biomarkers and immunosuppressive mechanisms in SCC.

## 2 Materials and Methods

### 2.1 Human Samples

Human cancer tissues (BCC, *n* = 5; SCCIS, *n* = 3; SCC, *n* = 3) were collected from skin cancer patients at the Department of Plastic and Reconstructive Surgery, Seoul National University Boramae Hospital (Institutional Review Board No. 30–2017‐11), using wide excision surgery. The wide excision was designed to include both the cancerous and adjacent skin tissues with a safety margin of 5 mm to 30 mm, varying by cancer type, and was completely excised. These freshly excised biopsies were split into two sections: one half of each sample was promptly dissociated into a single‐cell suspension for scRNA‐seq, and the other half was preserved in formalin for immunohistochemical analysis and Spatial Transcriptomics analysis. All diagnoses of cancer types were verified histologically by a board‐certified dermatopathologist.

### 2.2 Single‐Cell cDNA and Library Preparation

Single‐cell cDNA and library construction were performed following the 10 × Genomics guidelines (10 × Genomics 5′ v2, CG000331, Rev. D). Cells were captured using a Chromium controller (10 × Genomics) at the Seoul National University College of Medicine, Republic of Korea. Gel beads containing 10 × barcodes, UMI, and oligo dTs were used to form GEMs (Gel bead‐in EMulsions) with the cells. After generating GEMs, the captured mRNA underwent reverse transcription via PCR to produce cDNAs. During cDNA synthesis, each cDNA was tagged at the 5− end with a UMI and a barcode indicating its cell of origin. The full‐length cDNA was then amplified via PCR to generate sufficient mass for library construction and quantified using the ScreenTape on a 4150 TapeStation instrument (Agilent). Then, the libraries were sequenced on a NovaSeq6000 (Illumina).

### 2.3 Preprocessing of scRNA/TCR‐Seq Data

The FASTQ files generated from Illumina sequencing output were aligned to the human reference transcriptome (GRCh38) using 10× Genomics Cell Ranger v7.0.1. Raw expression data were loaded into Seurat (v4.9.9) R (4.2.1) for downstream analysis of our scRNA‐seq [15]. Background transcript contamination in retained true cells was eliminated using SoupX with the contamination fraction set to 20% (setContaminationFraction = 0.2) [16]. Doublets or multiplets were removed using scDblFinder with default parameters [17], and cells within each dataset were filtered to include only those with more than 300 genes and less than 10% of mitochondrial genes, resulting in a total of 117,663 cells: SCCIS (ID: SCCIS1, SCCIS2, SCCIS3; *n* = 19,085 cells, 4310 cells, and 13,269 cells, respectively), SCC (ID: SCC1, SCC2; *n* = 19,277 cells and 13,987 cells), and BCC (ID: BCC1‐5; total: 68,782 cells). To eliminate technical variations in samples derived from different experiments, 10 samples were merged and integrated to remove the batch effect by scVI [18]. Normalized expression, which was normalized using default parameters, was applied to detect highly variable genes (HVGs) using the Seurat algorithm. Each library was used as the batch key for calculating HVGs and the batch variable in the scVI modeling. Unsupervised clustering was conducted using the Louvain algorithm followed by visualization by Uniform Manifold Approximation and Projection (UMAP). The cluster markers were found using the FindAllMarkers function, and the cell types were manually annotated based on the markers. Gene signature was performed using the “GSVA” package with default parameters. The data visualization and statistical analysis were performed in the licensed version of GraphPad Prism v10 and R (4.2.1) along with the following packages: ggplot2, ggpubr (v0.6.0). All the statistical tests were two‐tailed, and a *p*‐value of 0.05 was considered significant.

### 2.4 GeoMx Digital Spatial Profiler (DSP) Library Preparation

Spatial transcriptomics was performed using the GeoMx DSP platform of the whole‐transcriptome atlas (NanoString Technologies, Seattle, WA, USA). Tissue sections from formalin‐fixed paraffin‐embedded (FFPE) blocks were mounted on a charged Leica Bond slide. Tissue slides were deparaffinized and subjected to antigen retrieval procedures and RNA hybridization according to the manufacturer's user manuals. These slides were stained with morphology markers containing SYTO13 (NanoString), PanCK (Novus, AE1 + AE3), CD68 (Santa Cruz, KP1), and CD3 (Novus, C3e/1308). The stained slides were loaded into the GeoMx DSP instrument. Next, selected regions of interest (ROIs) were chosen within the combined stroma/tumor areas to represent distinct sections of the tumor. These ROIs were then segmented by applying PanCK staining, which separated the tumor and stroma areas of illumination (AOI). Oligonucleotides from AOI were released upon exposure to ultraviolet light and collected. These oligonucleotides were amplified and finally pooled. The generated libraries were paired‐end sequenced on Illumina's NextSeq 6000.

### 2.5 Preprocessing of GeoMx DSP Data

The FASTQ files processed from the library were converted to Digital Count Conversion (DCC) files by NanoString's GeoMx NGS pipeline software (V.2.1). DCC files were imported back into the GeoMx DSP platform to generate an expression count matrix. After QC according to NanoString's recommendations, the GeoMx dataset was loaded into the StandR package (v1.9.0) for downstream analysis [19]. The segments were maintained with at least 100 “NucleiCount,” resulting in 16 segments, and 18,677 target genes were used for analysis. UMAP visualizations were generated after TMM normalization was applied to each ROI, and integration was performed to remove batch effects using RUV4 with default parameters in StandR. The data visualization and statistical analysis were performed in the licensed version of GraphPad Prism v10 and R (4.2.1) along with the ggplot2 package.

More materials and methods can be found online as Appendix S1.

## 3 Results

### 3.1 The Heterogeneity of Cellular Composition Between cSCC and BCC

**FIGURE 1:**
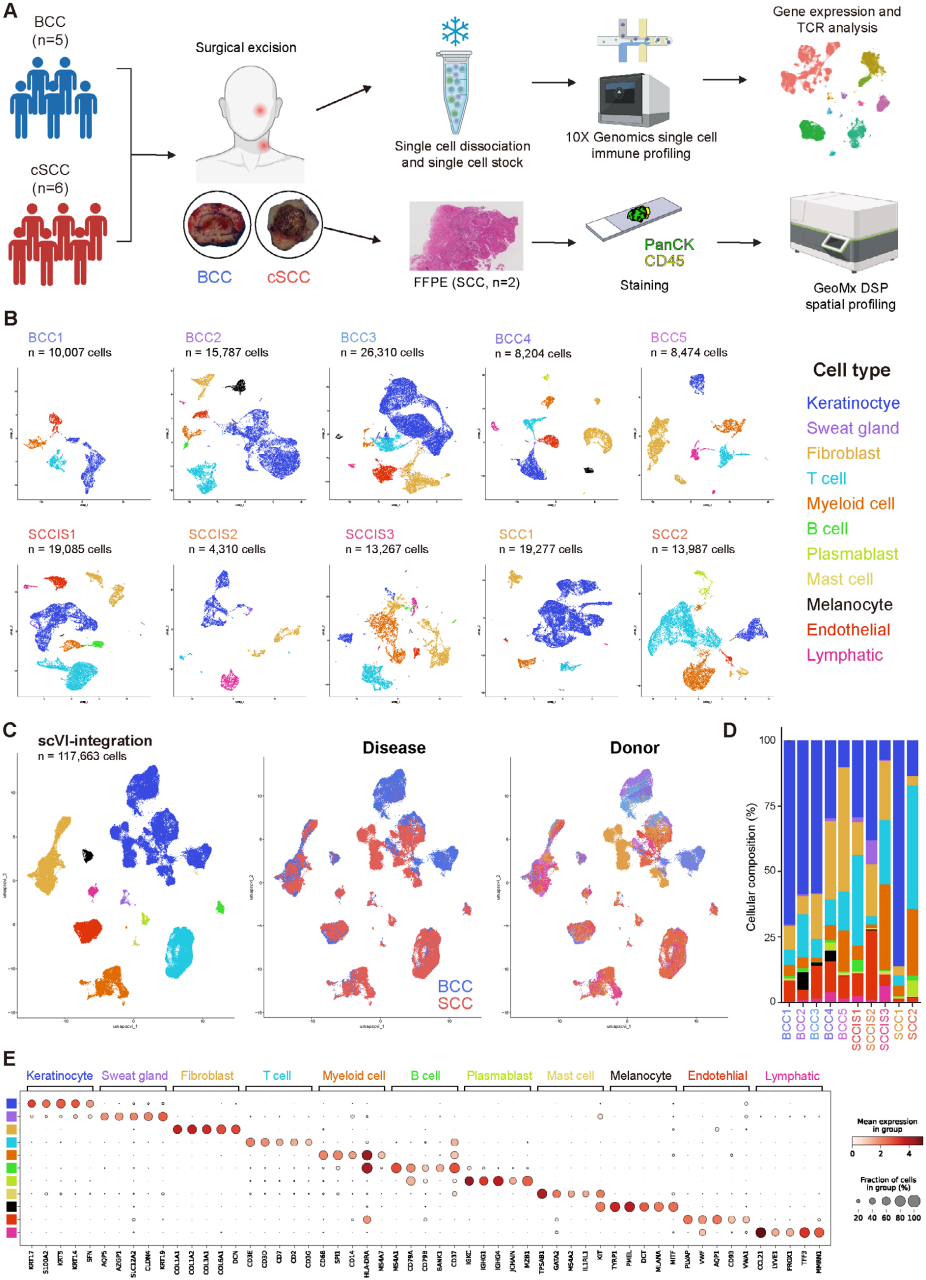
Cellular characterization SCC skin biopsy using scRNA‐seq. (A) Schematic representation of isolation and processing workflow from BCC and SCC tissue. (B) UMAP plot of skin samples from BCC biopsies (*n* = 5) and SCC biopsies (*n* = 5). IDs represent cancer type and donor. Eleven distinct meta‐clusters are defined on the right and color‐coded accordingly per cell type. (C) Clustering of integrated BCC and SCC datasets is grouped by meta‐clusters, diseases, and donor using scVI‐integration. Meta‐clusters, diseases, and donor are labeled and color‐coded. (D) Proportion of cell types grouped by donor. (E) Dot plot of top five marker genes identified by differential gene expression among cell types. White, low average gene expression; dark red, high average gene expression. Size of circle represents the percentage of cells expressing gene markers of interest.

We dissociated surgical biopsy samples taken from a cohort of patients diagnosed with BCC (*n* = 5) and cSCC (SCCIS, *n* = 3; SCC, *n* = 2) to acquire single‐cell suspensions and subjected them to the 10× Genomics Chromium platform. After the removal of doublets, ambient RNA, and dead cells, a total of 117,663 single cells were obtained (Figure 1A). Based on their cell‐specific markers, we identified 11 cell types, including Keratinocyte, Sweat gland, Fibroblast, T cell, Myeloid cell, B cell, Plasmablast, Mast cell, Melanocyte, Endothelial cell, and Lymphatic endothelial cell, respectively (Figure 1B) [12, 13]. To integrate the BCC and cSCC datasets, we used scVI [18]. In contrast to non‐epithelial cell types, keratinocytes exhibited distinct clustering patterns across tumor types and donors (Figure 1C,D). Comparative analysis of the proportion of each cell type revealed no significant differences between BCC and SCC (Data not shown).

### 3.2 Defining and Characterizing Malignant Epithelial Cells

**FIGURE 2:**
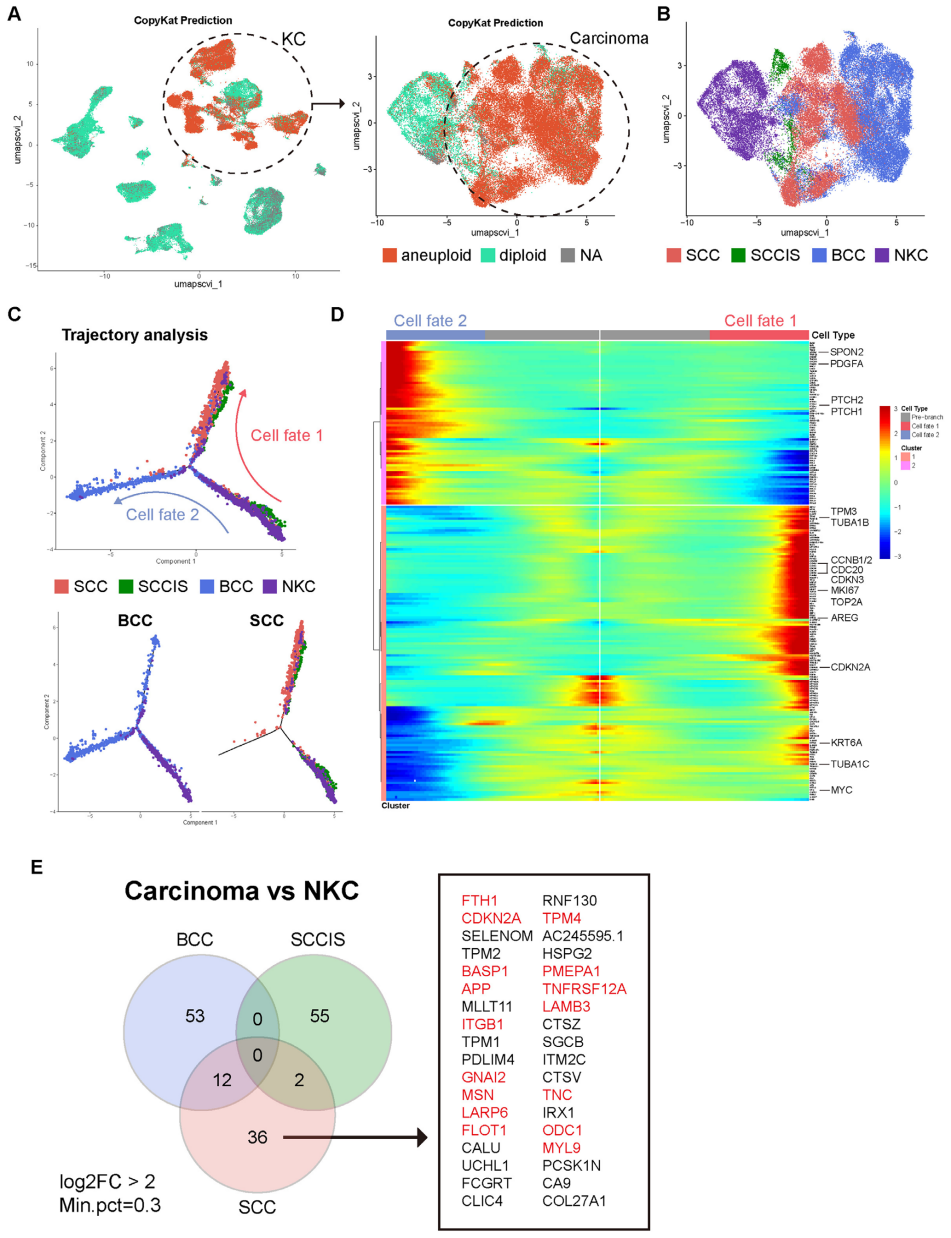
Analysis of SCC properties and cellular evolutionary trajectory. (A) The Copycat software identifies the malignant epithelial cells; aneuploidy: malignant; diploid: normal. The cells marked with circles are keratinocytes, while the aneuploid cells are labeled as carcinoma. (B) Clustering of normal and malignant epithelial cells grouped by diseases. (C) Upper, trajectory plot of keratinocyte. Arrows indicate the differentiation pathway and directions from normal keratinocyte to two cell fates; red: cell fate 1; blue: cell fate 2. Bottom, cells along the trajectory divided into two groups based on BCC and SCC samples. Diseases are labeled and color‐coded. (D) Heatmap depicting the key genes involved in branch determination and their functions. Heatmap showing the two dynamic gene expression patterns between two cell fates. In each cell fate, genes related to pathogenesis are indicated with larger font size. (E) Venn diagram of disease‐specific DEGs (versus normal keratinocyte, log2 fold‐change > 2, minimum fraction of cells > 0.3). The genes highlighted in red are associated with SCC growth and progression.

To define malignant cells from normal cells, we performed single‐cell CNV analysis by using “CopyKAT” package [20]. The result indicated a distinction between malignant and normal cells among the 117,663 cells and inferred that malignant cells belong to the keratinocyte (KC) population. Malignant KC (aneuploid, *n* = 43,866 cells), referred to as carcinoma, and normal KC (diploid, *n* = 10,382 cells) were successfully identified (Figure 2A). To define which skin cancer types the Carcinoma clusters originated from, we labeled carcinoma clusters according to cancer type as BCC (*n* = 23,842), SCCIS (*n* = 2659), and SCC (*n* = 15,365), along with normal keratinocytes (NKC) (Figures 2B and S1). Since BCC and cSCC originate from NKC, we utilized the “Monocle2” package to delineate their differentiation pathway. NKC were positioned at the initial point of the trajectory, subsequently branching into two distinct pathways referred to as cell fate 1 and cell fate 2. Remarkably, SCCIS and SCC resided at the final segment of trajectory 1, whereas BCC populated the endpoint of trajectory 2 (Figure 2C). We observed the accumulation of molecules related to BCC progression such as *SPON2*, and components of the hedgehog pathway, including *PDGFA*, *PTCH1*, and *PTCH2*, along trajectory 2. Conversely, *CDKN2A*, a molecule possibly associated with the development of cSCC, coincided with trajectory 1 [6, 9]. Additionally, several oncogenes (*MYC*, *TUBA1C*, and *TPM3*), cyclin‐dependent genes (*CCNB1*, *CDC20*, and *CDKN3*), and genes associated with proliferation (*MKI67* and *TOP2A*) were enriched in cSCC progression (Figure 2D). Moreover, to characterize genes during the evolution of these cancer types, we acquired the upregulated genes in BCC, SCCIS, and SCC compared to NKC using differentially expressed genes (DEGs) analysis (Figure 2E). As a result, we identified 36 SCC‐specific upregulated genes, of which 16 were highlighted. Consistent with previous findings, SCC exhibited increased *CDKN2A* compared to NKC [6, 9], and, as known, *TNC* and *LAMB3* associated with the PI3K/AKT/mTOR pathway were upregulated [21, 22]. Furthermore, six tumor progression genes (*GNAI2*, *ODC1*, *APP*, *TNFRSF12A*, *BASP1*, and *LARP6*) and nine migration and metastasis‐related genes (*MSN*, *FLOT1*, *LAMB3*, *MYL9*, *ITGB1*, *FTH1*, *TPM4*, and *PMEPA1*), which could explain the invasive characteristics of SCC, were identified. These genes represent potential key drivers of SCC development from NKC and serve as potential targets for the treatment of SCC.

### 3.3 Characteristics of SCC‐Specific COL6A1+/ITGA5+ Carcinoma

**FIGURE 3:**
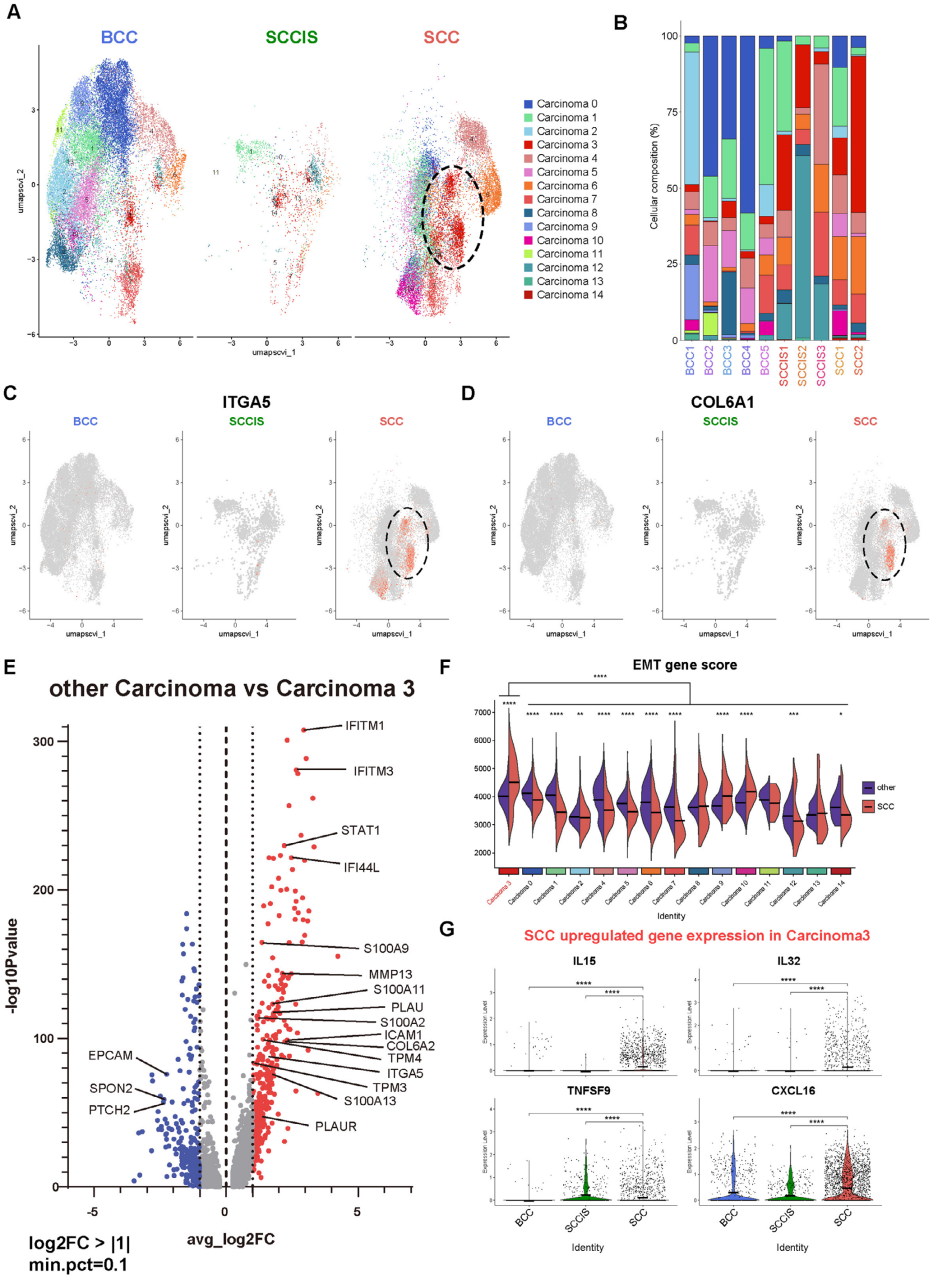
Characteristics of SCC‐specific carcinoma subpopulations. (A) UMAP for carcinoma cells grouped by sub‐clusters. Fourteen carcinoma cells were identified. Cells are color‐coded accordingly. (B) Proportion of cell types grouped by donor. (C, D) Feature plots of expression distribution for ITGA5 (C), and COL6A1 (D), among diseases. (E) Volcano plot of differentially expressed genes between carcinoma clusters and carcinoma 3. Log2 fold‐change is shown on the x‐axis and –log10 *p* values are shown on the y‐axis. A total of 353 genes were found to be increased in carcinoma 3 compared to other carcinomas. (F) Violin plots of the hallmark EMT gene signature score in Carcinoma subpopulations between SCC and other tumor. Carcinoma 3 in SCC exhibited the highest expression of Hallmark EMT gene signature (G) Violin plots showing SCC upregulated genes in carcinoma 3 grouped by diseases. The genes involved in attracting and interacting with immune cells were found to be increased. *p* < 0.05 suggested significant differences. ∗*p* < 0.05, ∗∗*p* < 0.01, ∗∗∗*p* < 0.001, ∗∗∗∗*p* < 0.0001 and ns, not significant.

To address which clones are associated with enhanced aggressive traits of SCC, we subclustered 43,886 malignant epithelial‐derived cells and identified 15 carcinoma cell clusters (Figure 3A). The proportion of Carcinoma 3 significantly increased within the carcinoma in cSCC compared to BCC (Figure 3B) with specific expression of *ITGA5* and *COL6A1* in SCC (Figure 3C,D). *ITGA5* has been previously reported as a marker of tumor‐specific keratinocytes (TSK) in SCC and associated with recurrence and migration [13, 14]. COL6A is a major component of the extracellular matrix (ECM), mainly found in the basement membrane region. Recently, *COL6A1* has been recognized as important for tumor growth and metastasis, and it has been reported to be expressed in a variety of cancers including cervical squamous cell carcinoma and pancreatic carcinoma [23, 24]. We next explored DEGs between Carcinoma 3 and other carcinoma clusters using volcano plot analysis. Carcinoma 3 exhibited increased expression of S100‐protein related genes (*S100A2, S100A9, S100A11*, and *S100A13*), interferon related genes (*STAT1, IFITM1, IFITM3*, and *IFI44L*), and metastasis related genes (*TPM3, TPM4, MMP13, PLAU*, and *PLAUR*) (Figure 3D). STAT1 promotes IFNAR and JAK pathway which potentially drives the invasion of cSCC [25]. S100A9 activates the NFkB signaling pathway, while S100A11 activates the mTOR pathway, inducing progression and invasion in cSCC [26, 27]. These findings indicate that Carcinoma 3 might have local invasive and metastatic potential. To evaluate the migration potential of Carcinoma 3, we performed EMT gene signature scoring using the Hallmark EMT gene set from MSigDB and observed that Carcinoma 3 exhibited the highest EMT activity. To further investigate this finding, we compared EMT scores between SCC and other cancer types, confirming that SCC tumors showed significantly elevated EMT gene scores (Figure 3E). Moreover, we identified disease‐specific upregulated genes in SCC, particularly in Carcinoma 3, and discovered immune cell migration and proliferation‐associated genes, such as *IL15*, *IL32*, *CXCL16*, and *TNFSF9* (Figure 3F) [28, 29]. These results indicate that the specific cluster identified in SCC drives the invasive characteristics of SCC and the recruitment of specific immune cells.

### 3.4 Identifying Tumor‐Associated CXCR6+ Tregs Populations

**FIGURE 4:**
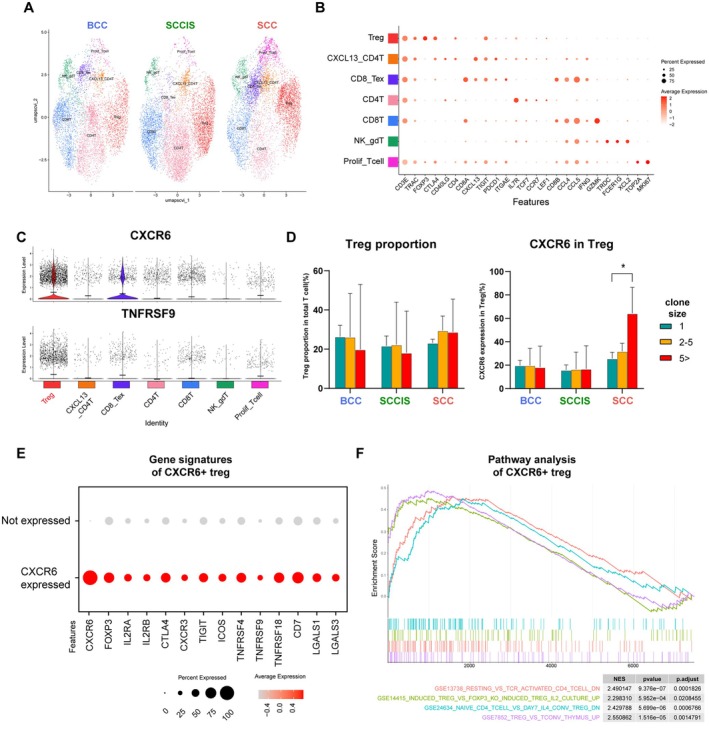
CXCR6 expressing Treg migrate to tumor site and exert immunosuppressive functions. (A) UMAP showing the distribution of T cell subsets. (B) Dot plot showing the highly expressed marker genes in each immune cell type. The dot color represents the average expression level of the marker genes in each cell type and the dot size represents the percentage of cells expressing the marker genes in each cell type. (C) Violin plots of expression distribution for TNFRSF9 and CXCR6 among diseases. Two genes were highlighted in Treg clusters where both genes were expressed. (D) Bar plot showing Treg proportion in T cells and CXCR6 expression in Treg grouped by disease and clonal size. In the analysis employing 2‐way ANOVA, an increase in the proportion of CXCR6 expression was observed in SCC during clonal expansion (* = 0.287). (E) Dot plot showing the expression levels of immunosuppressive gene signature genes in Treg grouped by CXCR6 expression levels. Treg expressing CXCR6 showed an increased immunosuppressive function. (F) Gene set enrichment analysis (GSEA) comparing CXCR6+ versus CXCR6‐ Tregs revealed enrichment of multiple immunologically relevant gene sets. Each line represents the running enrichment score across the ranked gene list for each gene set. The colored tick marks below indicate the positions of genes from each signature within the ranked list. Associated normalized enrichment score (NES), *p* values and adjusted *p* values are shown in the table.

To define tumor‐associated lymphocytes interacting with Carcinoma 3, we subclustered T lymphocytes based on the expression of *CD3D* and *TRAC*, yielding a total of 21,093 cells (n_BCC_ = 6191, n_SCCIS_ = 7981, and n_SCC_ = 6921) (Figure 4A). Based on well‐established markers, we identified seven T cell subpopulations: (1) Tregs (*FOXP3* ^+^, *CTLA4* ^+^, and *TIGIT* ^+^), (2) CXCL13_CD4T (*CD4* ^+^ and *CXCL13* ^+^), (3) CD8_Tex (*CD8A* ^+^, *TIGIT* ^+^, and *PDCD1* ^+^), (4) CD4T (*CD4* ^+^ and *IL7R* ^+^), (5) CD8T (*CD8* ^+^ and *GZMK* ^+^), (6) NK_gdT (*TRDC* ^+^ and *XCL2* ^+^), and (7) Prolif_Tcell (*MKI67* ^+^ and *TOP2A* ^+^) (Figure 4B) [30]. We next identified cells expressing the receptors *CXCR6* and *TNFRSF9*, which correspond to their ligands *CXCL16* and *TNFSF9* in Carcinoma 3, particularly in SCC (Figure 4C). CXCR6 serves as a central indicator for resident memory T (Trm) cells, contributing to immune surveillance by interacting with epithelial cells [31]. TNFRSF9 (commonly referred to as 4‐1BB or CD137) fosters T cell proliferation and enhances the immune‐suppressive functions of Tregs within tumors [32]. Both *CXCR6* and *TNFRSF9* are predominantly expressed in Tregs, suggesting the potential interaction of specific Tregs and Carcinoma 3. Within our dataset, no significant differences were observed in the proportion of Tregs across different cancers or clone sizes. However, the prevalence of *CXCR6* expression among Tregs was highest in SCC samples characterized by larger clone sizes (Figure 4D). Next, we examined the DEGs in Tregs based on CXCR6 expression. In CXCR6+ Tregs, we observed elevated expression of genes related to immune suppression such as *FOXP3*, *IL2RA, IL2RB*, *LGALS1*, and *LGALS3*, along with increased expression of costimulatory molecules like *TIGIT*, *CTLA4*, *ICOS*, *TNFRSF4*, *TNFRSF9*, and TNFRSF18 (Figure 4E) [33]. To further understand their functional state, we performed gene set enrichment analysis (GSEA) comparing CXCR6^+^ versus CXCR6^−^ Tregs. Notably, CXCR6^+^ Tregs were significantly enriched for gene sets associated with TCR‐activated CD4^+^ T cells (GSE13738) and activated or induced Treg signatures (e.g., GSE14415, GSE24634, and GSE7852) (Figure 4F). Taken together, these results imply that CXCR6^+^ clonally expanded Tregs in SCC may possess migratory properties and a transcriptional profile indicative of immunoregulatory activity, potentially contributing to the immunosuppressive tumor microenvironment.

### 3.5 Potential Receptor–Ligand Interaction Between Carcinoma 3 and Tregs

**FIGURE 5:**
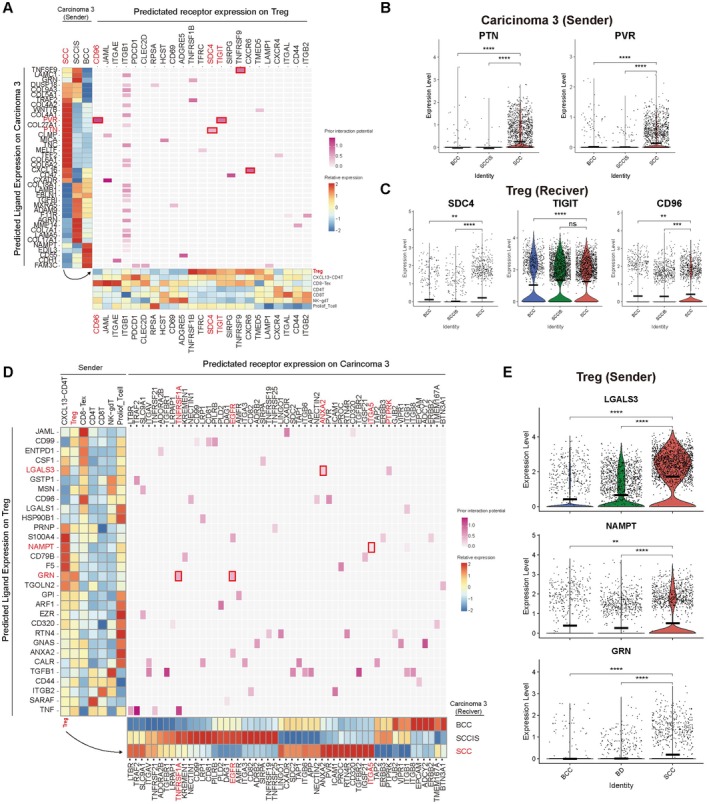
Cellular crosstalk between carcinoma 3 and Treg. (A) Left, heatmap of average log2 fold‐change across carcinoma among diseases of NicheNet top predicted ligands expressed by carcinoma 3. Bottom, heatmap of average log2 fold‐change of ligand‐matched receptors expressed by T cell subtype. Middle, heatmap of predicted ligand‐receptor interaction potential between carcinoma 3 and Treg. SCC‐specific interactions are indicated by red boxes. (B, C) Violin plot showing predicted SCC ligands in carcinoma 3 (B) and ligand‐matched receptors (C) grouped by diseases. Ligands that activate Treg specifically increased in SCC. (D) Left, heatmap of average log2 fold‐change across T cell subtypes of NicheNet top predicted ligands expressed by Treg. Bottom, heatmap of average log2 fold‐change of ligand‐matched receptor expressed in carcinoma 3 grouped by diseases. Middle, heatmap of predicted ligand‐receptor interaction potential between Treg and carcinoma 3. SCC‐specific interactions are indicated by red boxes. (E) Violin plot showing predicted ligands in Treg grouped by diseases. Ligands that promote cancer proliferation and metastasis specifically increased in SCC.

To identify communication between Carcinoma 3 and Tregs, we performed receptor–ligand interaction analysis among Carcinoma 3 and T cell populations using NicheNet. Consistent with a Carcinoma 3–T cell niche in SCC, robust signaling to Tregs was facilitated through multiple ligand–receptor interactions, encompassing TNFSF9‐TNFRSF9 and CXCL16‐CXCR6, as well as PTN‐SDC4, PVR‐CD96, and PVR‐TIGIT (Figure 5A). Carcinoma 3 specifically expressed PTN and PVR in SCC compared to BCC and SCCIS (Figure 5B). PTN is a heparin‐binding growth factor and has been reported to play an important role in cell–cell adhesion, cell motility, cell division, immune cell migration, and angiogenesis in various cancers [34, 35]. PVR predominantly interacts with TIGIT and is likely to foster an immunosuppressive phenotype in cells expressing TIGIT [36]. PVR is known for its ability to attach to CD96, which triggers an immunosuppressive reaction in mouse models. However, a direct inhibitory effect through CD96 has yet to be established in human research [37]. These receptors, identified in Tregs, were also significantly upregulated in SCC (Figure 5C). Conversely, ligand–receptor pairs including *LGALS3*‐*ANXA2*, *NAMPT*‐*ITGA5*, and *GRN*‐*TNFRSF1A* were identified in Tregs (Figure 5D). LGALS3 promoted tumor progression, invasiveness, and immune suppression, and blocking LGALS3‐ANXA2 has been documented to initiate cell apoptosis by suppressing all downstream EGFR signaling pathways [38]. NAMPT/Visfatin can modulate various downstream signaling pathways, including AKT/PI3K and ERK/MAPK, potentially increasing cell survival and proliferation in cSCC [39]. GRN overactivity occurs in many types of cancers and imparts an aggressive phenotype on poorly tumorigenic carcinomas [40]. Overall, our findings suggest that the SCC‐specific carcinoma cluster may engage in transcriptionally inferred interactions with Tregs, potentially contributing to an immunosuppressive microenvironment that supports tumor progression.

### 3.6 Spatial Transcriptomic Analysis Supports Enrichment of Carcinoma 3 at the Tumor–Immune Boundary

**FIGURE 6:**
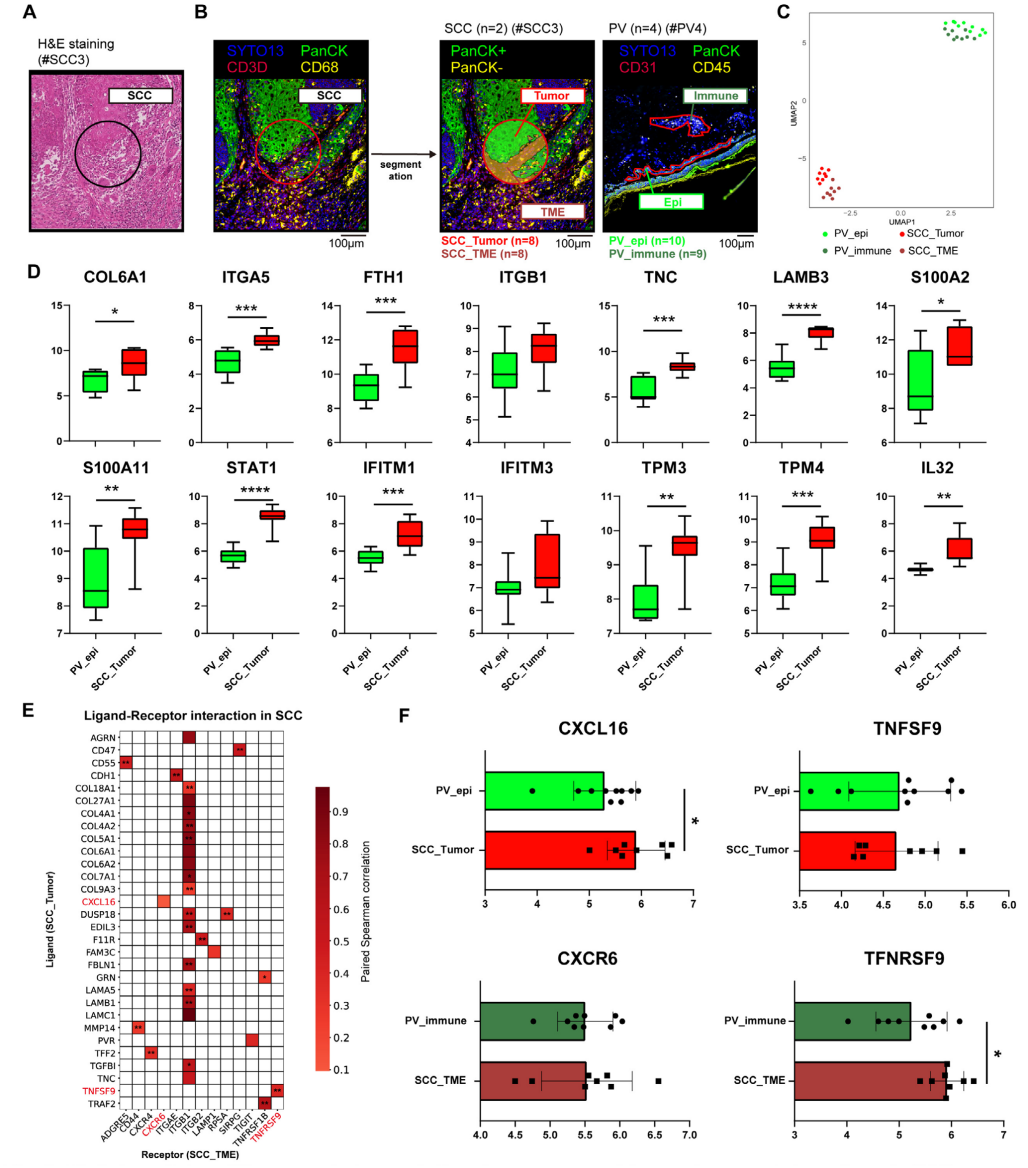
Digital spatial profiling reveals transcriptional programs and tumor–immune interactions in SCC. (A) Example of a representative SCC sample stained with H&E. Inset shows a higher magnification of a selected ROI. (B) Representative GeoMx spatial transcriptomics images from squamous cell carcinoma (SCC), and pemphigus vulgaris (PV) skin lesions. In SCC samples (*n* = 2), regions of interest (ROIs) were selected to capture PanCK^+^ tumor areas in close proximity to immune cell infiltrates. ROIs were segmented into SCC_Tumor and SCC_TME based on PanCK expression. In PV and PSO lesions, epithelial and immune compartments were delineated using CD45/CD31 and PanCK markers. (C) UMAP plot displaying spatial transcriptomic profiles from SCC (*n=*8 tumor, *n* = 8 TME), and PV (*n* = 10 epithelial, *n* = 9 immune) regions. Each point represents an individual area of interest (AOI), color‐coded by lesion type and tissue compartment. (D) Boxplots illustrating genes associated with SCC progression and previously identified in the Carcinoma 3 cluster, the majority of which were significantly upregulated in SCC_Tumor compared to PV and PSO epithelial regions. (E) Heatmap showing paired Spearman correlation analysis of ligand–receptor gene pairs in SCC. Each row represents a ligand expressed in SCC_Tumor regions, and each column represents its corresponding receptor in SCC_TME regions. Ligand–receptor pairs were pre‐selected based on top‐ranked interactions predicted from Carcinoma 3 in Figure 5A, and only those with Spearman correlation coefficient ≥ 0 are shown. Cell color represents the strength of correlation, and values within each cell indicate the associated *p* value. The red‐highlighted ligand–receptor pairs were previously identified as key components of the Carcinoma 3—Treg interaction network. (F) Scatter plots showing the expression of selected ligands (CXCL16, TNFSF9) and their corresponding receptors (CXCR6, TNFRSF9) in SCC and PV samples. Ligands were measured in SCC_Tumor and PV epithelial regions, while receptors were measured in SCC_TME and PV immune regions. Among these, CXCL16 and TNFRSF9 showed significant upregulation in SCC samples. *p* < 0.05 suggested significant differences. ∗*p* < 0.05, ∗∗*p* < 0.01, ∗∗∗*p* < 0.001, ∗∗∗∗*p* < 0.0001 and ns, not significant.

To examine the localization of Carcinoma 3 in SCC specimens from a spatial perspective, we performed spatial transcriptome analysis on SCC samples (SCC1 and SCC3) using the NanoString GeoMx Digital Spatial Profiler (DSP) platform (Figure 1A). Based on histological features and immunostaining for SYTO13, PanCK, CD3D, and CD68, we selected eight regions of interest (ROIs) and defined segments representing tumor cells (SCC_Tumor, PanCK+, *n* = 8) and the surrounding TME (SCC_TME, PanCK‐, *n* = 8) (Figure 6A,B). These ROIs were selected based on the presence of PanCK^+^ tumor regions directly adjacent to dense CD3D^+^ and CD68^+^ immune infiltrates, identified through both H&E and multiplex immunofluorescence staining, to capture tumor margins involved in immune interaction. To compare SCC‐specific transcriptomic characteristics with a nonmalignant inflammatory skin condition, we utilized internal GeoMx data from pemphigus vulgaris (PV) [41], which is a keratinocyte‐derived autoimmune disease characterized by strong immune cell infiltration and intraepidermal blistering. We included PV regions of interest (ROIs) categorized as either PanCK^+^ (PV_epi, *n* = 10) or PanCK^−^ (PV_immune, *n* = 9), analogous in compartment identity to the SCC_Tumor and SCC_TME groups. All 37 AOIs (SCC and PV) passed quality control and were included in downstream analyses. UMAP analysis demonstrated a clear separation between SCC and PV samples, indicating distinct transcriptomic profiles between malignant and nonmalignant epithelial inflammation (Figure 6C). We compared the expression of Carcinoma 3‐associated genes between SCC_Tumor and PV epithelial regions (PV_epi) (Figure 6D). Genes such as COL6A1, ITGA5, FTH1, TNC, LAMB3, S100A2, S100A11, STAT1, IFITM1, TPM3, TPM4, and IL32 were significantly upregulated in SCC_Tumor compared to PV_epi. To further investigate how Carcinoma 3 may participate in spatially coordinated tumor–immune interactions, we evaluated ligand–receptor gene pairs between SCC_Tumor and SCC_TME regions. For each ligand–receptor pair preselected from the NicheNet analysis (Figure 5A), we calculated the paired Spearman correlation coefficient across spatially matched SCC_Tumor and SCC_TME segments (*n* = 8 AOI pairs) (Figure 6E). Several ligand–receptor pairs exhibited relatively strong correlations, suggesting spatially organized interactions between tumor and immune compartments. Among immune‐related interactions, TNFSF9–TNFRSF9 displayed a strong spatial correlation. Moreover, we compared multiple ligand–receptor pairs highlighted within the Carcinoma 3–Treg niche in SCC (Figure 6F). To validate the spatial association between Carcinoma 3 cells and CXCR6^+^ Tregs at the protein level, we performed multiplex immunofluorescence staining on serial sections of SCC (Figure S2). ITGA5^+^ PanCK^+^ tumor cells, corresponding to the Carcinoma 3 cluster, were localized at the tumor–stroma boundary, and CXCR6^+^ FOXP3^+^ CD3D^+^ regulatory T cells were observed in close proximity within the adjacent stroma. These findings provide direct spatial evidence supporting the Carcinoma 3–Treg niche and reinforce the transcriptionally inferred ligand–receptor interactions, suggesting that Carcinoma 3 cells at the tumor edge may actively shape the local immune landscape.

## 4 Discussion

The inter‐ and intra‐tumor heterogeneity of NMSC hampers the general interpretation of tumor progression and the shaping of the tumor microenvironment. However, current research on NMSC has primarily focused on single cancer types, resulting in a lack of comprehensive explanations regarding general observation and tumor progression. Therefore, we conducted the comparative analysis of BCC and cSCC using scRNA‐seq, which elucidated general principles and fostered a deeper, more specific understanding of each cancer type.

To understand how individual tumor cells shape the tumor microenvironment, we need to separate the tumor clones from the normal tissue cells from which they originated. However, distinguishing keratinocyte‐derived carcinoma from human skin was challenging due to the expression of some oncogenes in normal cells as well. To address this issue, we estimated copy number variation driving clonal evolution of tumor cells and distinguished cancer cells from normal cell types using the “CopyKAT” package. We successfully annotated cancer cells, enabling us to conduct trajectory analysis from normal to tumor cells. Our results confirmed that BCC and cSCC undergo different evolution processes. In BCC, we were able to confirm a BCC‐specific gene expression pattern, including *BCAM* and *EPCAM*, as identified in previous single‐cell studies [12]. Moreover, while mutations in the *PTCH* gene are known to occur in familial BCC, our results also demonstrated an increase in PTCH‐related genes in sporadic BCC. Conversely, in cSCC, we observed an increase in predominantly proliferative characteristics and confirmed that SCCIS and SCC share a common lineage. To understand which gene expressions influence the malignancy of SCC during its progression from SCCIS to SCC, we conducted a comparative analysis of cancer clones from SCC and SCCIS. Through this analysis, we defined 16 SCC‐specific genes, including *FTH1, CDKN2A, BASP1, APP, ITGB1, GNAI2, MSN, LARP6, FLOT1, TPM4, PMEPA1, TNFRSF12A, LAMB3, TNC, ODC1*, and *MYL9*, which are related to tumor progression and migration. This finding aligns with the known invasive characteristics of SCC, suggesting that these genes could serve as potential biomarkers or therapeutic targets for SCC.

Next, we performed subclustering of tumor cells to understand intra‐tumor heterogeneity. Interestingly, the Carcinoma 3 cluster with high expression of *COL6A1* and *ITGA5* was predominantly observed in SCC. *COL6A1* encodes the α1 chain of type VI collagen, a fundamental ECM protein essential for maintaining the structural integrity of various tissues. Recent studies indicate that COL6A1 is involved in several biological functions including cell migration, differentiation, wound healing, and preserving cell stemness [42]. Furthermore, increased expression of COL6A1 has been linked to enhanced motility and metastasis in lung, pancreatic, and cervical cancer cells, whereas reducing COL6A1 expression has been associated with decreased metastatic capabilities [23, 24]. *ITGA5* primarily serves as a receptor for fibronectin, partnering with integrin beta 1 to form a heterodimer. Research has established that ITGA5 is crucial for cancer cell proliferation, migration, invasion, and metastasis [43, 44]. Collectively, we propose that *COL6A1* and *ITGA5* expression in SCC enhances adhesion to the ECM, thereby promoting the malignancy of the cancer. Notably, the COL6A1+/ITGA5+ malignant epithelium cluster (Carcinoma 3) exhibited increased expression of *IL15*, IL32, *CXCL16*, and *TNFSF9*. IL15, IL32, and TNFSF9 have been reported to promote the survival and function of T cells and NK cells. CXCL16 induces immune cell homing and accumulation. These findings suggest that Carcinoma 3 in SCC leads to increased immune cell infiltration, thereby characterizing SCC as a hot tumor.

To determine which immune cells interact with Carcinoma 3, we performed subclustering of immune cells. In SCC, we observed increased expression of *CXCR6*, the receptor for Carcinoma 3's CXCL16, specifically in Tregs. This suggests a new potential migration mechanism of Tregs to the tumor site in SCC. Interestingly, among Tregs, those expressing *CXCR6* showed increased levels of functional markers such as *FOXP3*, *IL2RA*, *IL2RB*, and *CD7*, as well as immune checkpoint proteins, including *CTLA4*, *TIGIT*, and *ICOS*, and Tregs activity‐associated genes such as *TNFRSF4*, *TNFRSF9*, and *TNFRSF18* [33]. These findings indicate that although Carcinoma 3 in SCC recruits diverse immune cells, since it also drives the migration of functional Tregs via the CXCL16/CXCR6 axis, thereby creating a tumor microenvironment favorable for their survival. To further characterize the cellular communication between Carcinoma 3 and Tregs in SCC, we utilized the “NicheNet” package and focused SCC‐specific receptor–ligand pairs. We identified three ligands within Carcinoma 3 in SCC involving *TNFSF9*, *PTN*, and *PVR*, which promote the activation of Tregs [34, 35, 36]. This suggests that Carcinoma 3 in SCC not only promotes the migration of Tregs to tumor site but also enhances their immunosuppressive capabilities. Additionally, we focused the ligands of Tregs in the SCC niche. We confirmed increased levels of *LGALS3*, *NAMPT*, and *GRN* in Tregs within SCC. These pathways induce cancer proliferation, progression, and invasion [1, 38, 39, 40, 45]. This implies a novel mechanism whereby certain SCC clusters trigger Treg migration and activation, thus fostering an immunosuppressive microenvironment and SCC progression.

To investigate whether our observations from the scRNA‐seq study were being recapitulated and to explore the spatial locations of the Carcinoma 3 cluster in target tissues, we performed ST analysis on SCC slides from independent patients. To specifically assess Carcinoma 3 at the tumor–immune boundary, we selected regions of interest (ROIs) enriched for PanCK^+^ tumor areas adjacent to immune cell infiltrates based on histology and marker expression. First, using the GeoMx DSP (NanoString platform), we compared the genes related to Carcinoma 3 discovered by scRNA‐seq between SCC and PV to determine whether these tumor‐associated transcriptional programs could be distinguished from nonmalignant epithelial inflammation. We observed that the expression of *COL6A1* and *ITGA5*, as well as *FTH1*, *TNC*, *LAMB3*, *S100A9*, *S100A11*, *STAT1*, *IFITM1*, *TPM3*, and *TPM4*, which were expected to influence the progression of SCC, was significantly increased in SCC. These data suggest that Carcinoma 3 is preferentially located at the tumor–immune boundary and may possess invasive characteristics. Although COL6A1 was not clearly detected in tumor regions by immunofluorescence, its expression was consistently enriched in Carcinoma 3 across scRNA‐seq and spatial transcriptomic data. The strong fluorescence signal in the surrounding stroma may have masked or diluted the detection of COL6A1 specifically in tumor areas. Additionally, we focused on ligand–receptor interactions predicted from NicheNet analysis of SCC–Treg communication. The expression of *CXCL16*, a chemokine implicated in Treg migration, was significantly upregulated in SCC_Tumor regions. Although its corresponding receptor *CXCR6* was not differentially expressed, the *CXCL16*–*CXCR6* pair exhibited a positive spatial correlation, suggesting potential coordination between tumor cells and neighboring Tregs. In contrast, *TNFSF9* expression levels remained comparable between SCC and control samples, but its receptor *TNFRSF9* was markedly elevated in the TME and showed a significant correlation with *TNFSF9*, indicating spatial co‐regulation. These findings suggest that Carcinoma 3 may contribute to immune evasion in SCC by engaging ligand–receptor signaling pathways associated with Treg recruitment and activation. This potential interaction is further supported by spatial proximity between Carcinoma 3 cells and CXCR6^+^ regulatory T cells, as shown in serial immunofluorescence staining (Figure S2). In summary, we propose that Carcinoma 3, a tumor subpopulation enriched at the tumor–immune boundary, facilitates the formation of an immunosuppressive microenvironment. Furthermore, specific interaction between the SCC and Treg axis could potentially lead to the development of novel therapeutics for SCC.

## Author Contributions

**Hyun Seung Choi:** data curation (lead), formal analysis (lead), investigation (equal), visualization (lead), writing – original draft (lead). **Sunyoung Jung:** conceptualization (equal), investigation (lead), validation (lead), writing – original draft (equal). **Ki‐Myo Kim:** investigation (equal). **Mihyun Lee:** investigation (equal). **Jun Ho Park:** investigation (equal). **Sanha Hwang:** investigation (equal). **Seung Min Cha:** investigation (equal). **Jade N. Young:** writing – review and editing (equal). **Dina Poplausky:** investigation (equal). **Hyunsung Nam:** writing – review and editing (equal). **Nicholas Gulati:** writing – review and editing (equal). **Chung‐Gyu Park:** writing – review and editing (equal). **Hyun Je Kim:** conceptualization (equal), funding acquisition (equal), investigation (equal), supervision (equal), validation (supporting), writing – review and editing (lead). **Ji‐Ung Park:** conceptualization (equal), funding acquisition (lead), investigation (equal), supervision (equal), writing – review and editing (lead).

## Disclosure

Integration analysis of scRNA‐seq and ST of NMSC reveals mechanisms by which specific SCC clusters promote metastasis and shape an immunosuppressive TME, providing new approaches for targeted therapies to prevent SCC progression.

## Ethics Statement

This study was approved by the institutional review boards at the Seoul National University Boramae Hospital (IRB number: 30–2017‐11).

## Consent

Consent obtained directly from patient(s).

## Conflicts of Interest

The authors declare no conflicts of interest.

## Supporting information


Appendix S1.

Figure S1.

Figure S2.

## Data Availability

The data generated in this study are available upon request from the corresponding author.
