# Supplementary material for: CXCL16 Producing Tumor Clones Are Shaping Immunosuppressive Microenvironment in Squamous Cell Carcinoma via CXCR6 Regulatory T Cell
Source: Cancer Med. 2025 Aug 7;14(15):e71060. doi: 10.1002/cam4.71060 (PMC12331524; doi:10.1002/cam4.71060)
Supplement: Supplementary file 1 — Appendix S1. [file CAM4-14-e71060-s003.docx]

**Appendix S1. Supplementary Materials and Methods**

**S1.1 Copy number variation estimation.**

To further explore the tumor epithelial cell subpopulations in BCC, SCCIS, and SCC, total cells in each library were extracted. Aneuploid cells were identified using the CopyKAT v1.1.0 package with default parameters, and epithelial cells were re-clustered to obtain subsets of aneuploid carcinoma tumor clones. [1]

**S1.2 Construction of single-cell trajectory.**

Pseudotime analysis was performed with Monocle2 (v2.26.0) to infer the potential trajectory of epithelial and cancer cells.[2] the normalized data and clusters from the epithelial cells calculated in Seurat was passed directly into Monocle2. Pseudotime and cell states were inferred using orderCells function in monocle2, incorporating cluster identification from Seurat for consistency. Cell trajectories were visualized using the plot_cell_trajectory function in the Monocle2 package. The critical role of various gene series in the differentiation process were highlighted by additional analysis with Monocle2's plot_pseudotime_heatmap function.

**S1.3 Ligand-Receptor interaction mapping**

NicheNet analysis was performed as published code (nichenetr/seurat_wrapper_circos.md at master · saeyslab/nichenetr · GitHub).[3] Briefly, the expression data of interacting cells was extracted from Seurat object. NicheNet analysis and differential expression was performed using standard scripts with carcinoma clusters and T cell clusters. Therefore, the condition of interest was set to SCC, and the reference was set to BCC and SCCIS. Utilizing a model of already published ligand-target, ligand-receptor network and weighted integrated networks, NicheNet analysis is performed according to the published workflow.

To further prioritize spatially relevant ligand–receptor interactions, we extracted normalized expression matrices of SCC_Tumor and SCC_TME regions from the GeoMx dataset. For each ligand–receptor pair, we computed the paired Spearman correlation coefficient across matched tumor and TME AOIs (n = 8). Only interactions with a correlation coefficient ≥ 0.1 were retained for visualization.

**S1.4 multiplex fluorescence validation**

 To validate the spatial localization of tumor and regulatory T cell populations observed in scRNA-seq and spatial transcriptomic analyses, multiplex immunofluorescence staining was performed on FFPE tissue sections (5 μm) mounted on charged Leica BOND Plus slides. Sections were baked at 60 ℃ for 30 min, deparaffinized using CitriSolv, and rehydrated using ethanol and 1× phosphate-buffered saline. Antigen retrieval was performed using 1× Tris–EDTA buffer (pH 9.0) at 100 °C for 20 minutes. After washing in 1× PBS, tumor-target slides were stained with SYTO13 (NanoString, 121300303), PanCK (Novus, NBP2-33200, AF532), Integrin-α5 (Abcam, ab207269), and COL6A1 (Abcam, ab200430), and Treg-target slides were stained with SYTO13 (NanoString, 121300303), CD3E (Novus, NBP2-54392, AF647), FOXP3 (Invitrogen, 58-4776-42), and CXCR6 (Abcam, ab8023) for 1 hour at room temperature. The CXCR6 antibody was conjugated with Alexa Fluor® 594 using the Lightning-Link® Conjugation Kit (Abcam, ab269822), following the manufacturer's instructions. Slides were then scanned using the GeoMx DSP instrument in image-only mode for spatial validation, without transcriptomic profiling.

**S1.5 Statistical analysis**

For statistical comparisons between the two groups, we employed the Student t test or the Wilcoxon test or Mann-Whitney test. For comparisons of means among multiple groups, a one-way ANOVA was used. The proportions of Treg among different groups were determined using the two-way ANOVA test. All statistical analyses were performed using GraphPad prism v10 and R (4.2.1). P < 0.05 suggested significant differences. ∗, P < 0.05; ∗∗, P < 0.01; ∗∗∗, P < 0.001; ∗∗∗∗, P < 0.0001; ns, not significant.

**Reference**

[1] R. Gao, S. Bai, Y.C. Henderson, Y. Lin, A. Schalck, Y. Yan, T. Kumar, M. Hu, E. Sei, A. Davis, F. Wang, S.F. Shaitelman, J.R. Wang, K. Chen, S. Moulder, S.Y. Lai, N.E. Navin, Delineating copy number and clonal substructure in human tumors from single-cell transcriptomes, Nat Biotechnol, 39 (2021) 599-608.

[2] X. Qiu, Q. Mao, Y. Tang, L. Wang, R. Chawla, H.A. Pliner, C. Trapnell, Reversed graph embedding resolves complex single-cell trajectories, Nat Methods, 14 (2017) 979-982.

[3] R. Browaeys, W. Saelens, Y. Saeys, NicheNet: modeling intercellular communication by linking ligands to target genes, Nat Methods, 17 (2020) 159-162.
