# Supplementary figures and images for: CXCL16 Producing Tumor Clones Are Shaping Immunosuppressive Microenvironment in Squamous Cell Carcinoma via CXCR6 Regulatory T Cell
Source: Cancer Med. 2025 Aug 7;14(15):e71060. doi: 10.1002/cam4.71060 (PMC12331524; doi:10.1002/cam4.71060)

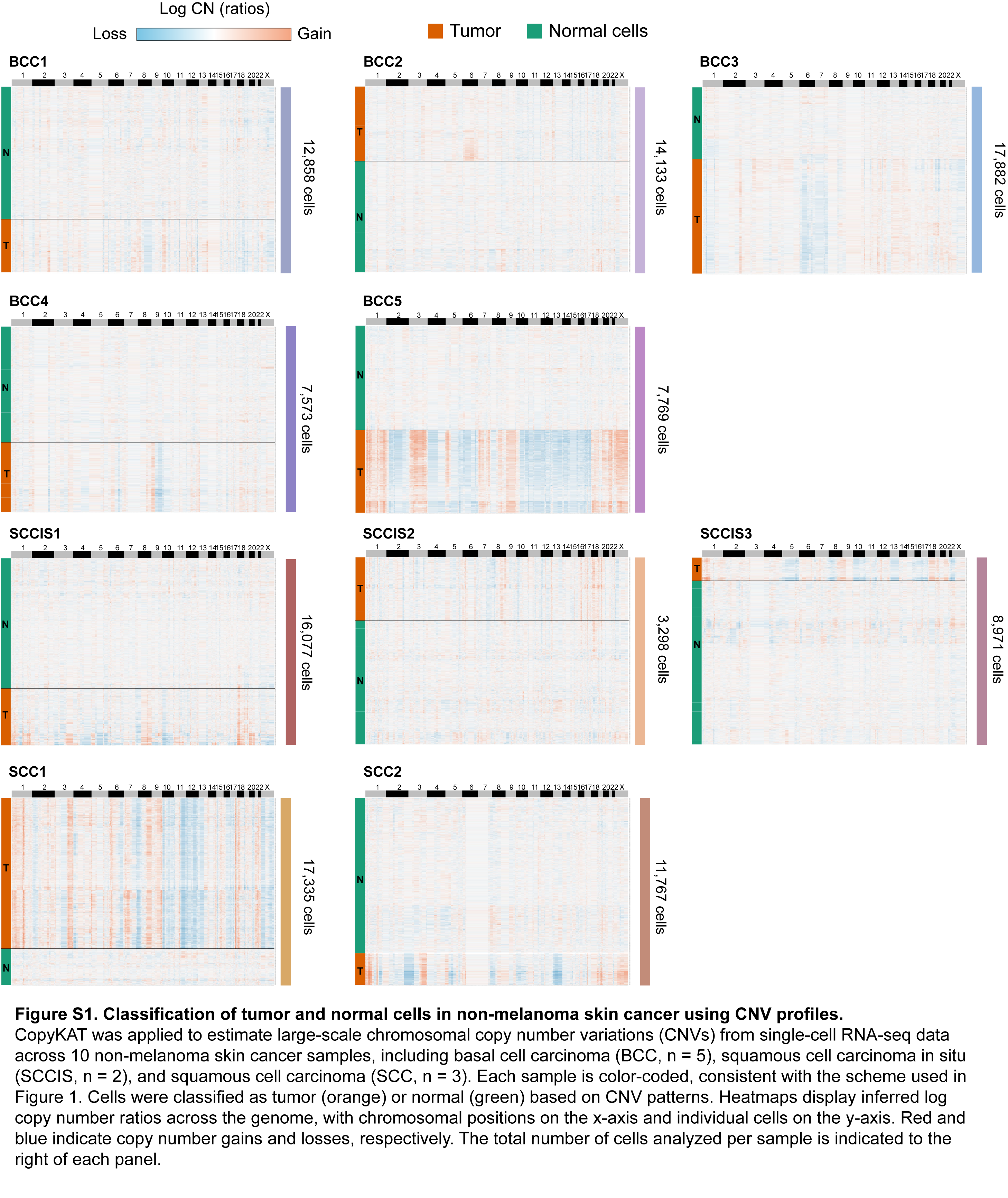

Supplement: Supplementary file 2 — Figure S1. [file CAM4-14-e71060-s002.tif]

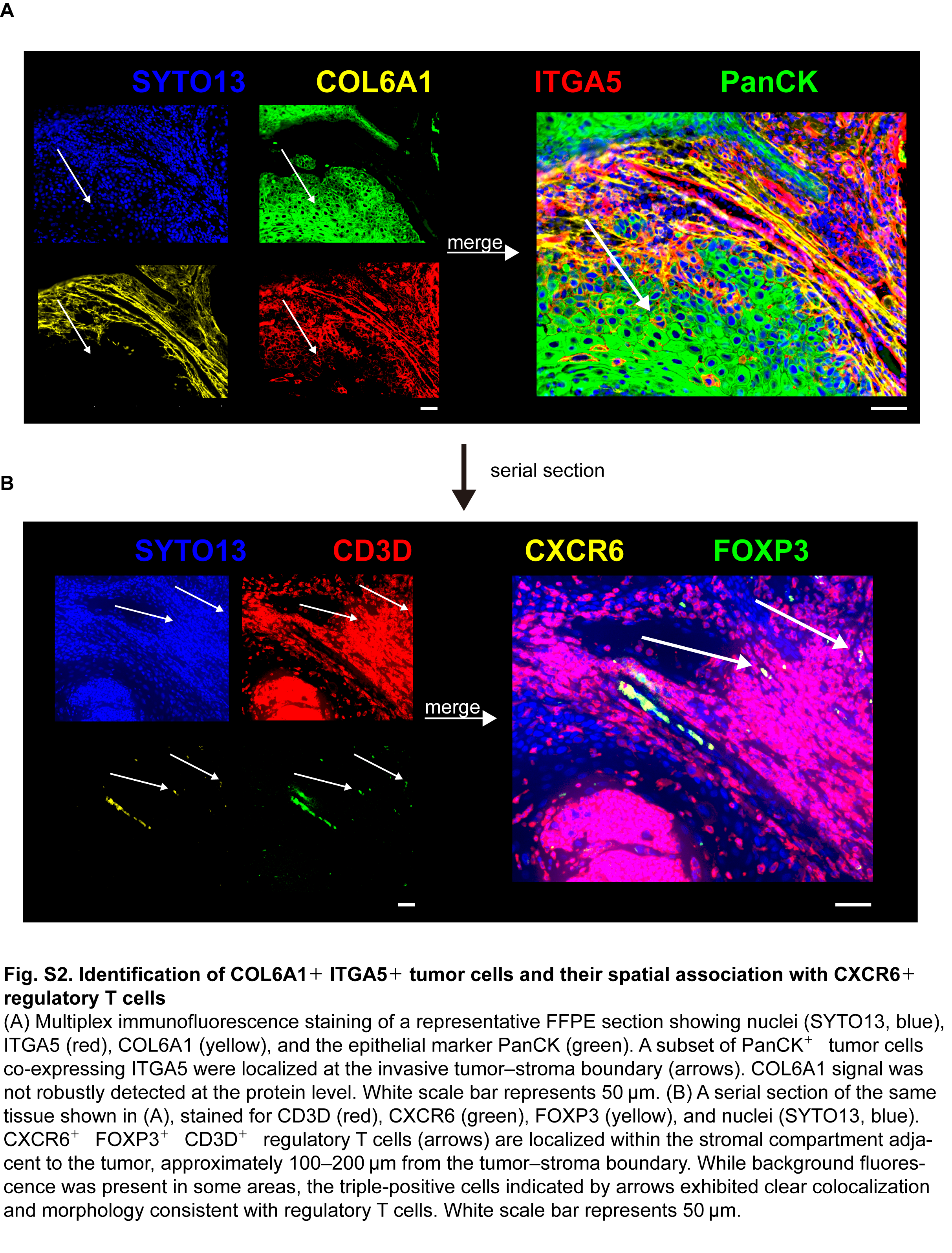

Supplement: Supplementary file 3 — Figure S2. [file CAM4-14-e71060-s001.tif]
